# Effect of Heterologous Vaccination Regimen with Ad5-nCoV CanSinoBio and BNT162b2 Pfizer in SARS-CoV-2 IgG Antibodies Titers †

**DOI:** 10.3390/vaccines10030392

**Published:** 2022-03-03

**Authors:** Maria Elena Romero-Ibarguengoitia, Diego Rivera-Salinas, Yodira Guadalupe Hernández-Ruíz, Ana Gabriela Armendariz-Vázquez, Arnulfo González-Cantú, Irene Antonieta Barco-Flores, Rosalinda González-Facio, Laura Patricia Montelongo-Cruz, Gerardo Francisco Del Rio-Parra, Miguel Ángel Sanz-Sánchez

**Affiliations:** 1 Research Department, Hospital Clínica Nova, San Nicolás de los Garza 66450, NL, Mexico; diego.rivera@udem.edu (D.R.-S.); yodira.hernandez@udem.edu (Y.G.H.-R.); ana.armendariz@udem.edu (A.G.A.-V.); c.argoca@novaservicios.com.mx (A.G.-C.); c.ibarco@novaservicios.com.mx (I.A.B.-F.); rgonzalezf@novaservicios.com.mx (R.G.-F.); laura.montelongo@udem.edu (L.P.M.-C.); gerardo.delrio@udem.edu (G.F.D.R.-P.); msanzs@novaservicios.com.mx (M.Á.S.-S.); 2 Vicerrectoría de Ciencias de la Salud, Escuela de Medicina, Universidad de Monterrey, San Pedro Garza García 66238, NL, Mexico

**Keywords:** COVID-19, coronavirus, immunization, antibodies, adverse events

## Abstract

The efficacy of one dose Ad5-nCoV has been concerning. This study aimed to evaluate the effect of a single dose BNT162b2 in individuals after a completed Ad5-nCoV vaccination regiment compared to a group without this boost measuring SARS-CoV-2 Spike 1–2 IgG antibodies in plasma. This observational study included a subgroup analysis of patients who were immunized with Ad5-nCoV in a northern city of Mexico. During follow-up, some patients self-reported having received a BNT162b2 booster. We report baseline IgG levels, 21–28 days after the Ad5-nCoV dose, three months, and an additional 21–28 days after BNT162b2 (four months after Ad5-nCoV). Seventeen patients, age 40 (16), 52.9% men, were analyzed. We created four groups: G1 and G2 refer to patients without a history of SARS-CoV-2 infection, vaccinated with Ad5-nCoV and Ad5-nCoV/BNT162b2 (*n* = 4 and *n* = 6), respectively; G3 and G4 included patients with a history of SARS-CoV-2 infection and immunized with Ad5-nCoV and Ad5-nCoV/BNT162b2 (*n* = 5 and *n* = 2), respectively. The Ad5-nCoV/BNT162b2 protocol reported higher antibody titers after 21–28 days. Median (IQR) values were: G1 46.7 (-), G2 1077.5 (1901), G3 1158.5 (2673.5), and G4 2090 (-) (*p* < 0.05). Headache and pain at injection site were the most frequent adverse reactions associated with Ad5-nCoV (*n* = 10, 83%) and BNT162b2 (*n* = 5, 83.3%), respectively. Patients receiving BNT162b2 after Ad5-nCoV had higher SARS-CoV-2 spike 1–2 IgG antibody titers and had no severe adverse reactions.

## 1. Introduction

On 11 March 2020, The World Health Organization (WHO) declared the SARS-CoV-2 pandemic due to the increasing number of cases of atypical pneumonia caused by a novel viral agent named Severe Acute Respiratory Syndrome Coronavirus-2 (SARS-CoV-2) [1]. This disease has had an overwhelming impact on health services. Most hospitals and health centers were overloaded due to the lack of a definitive and complete treatment against this agent, as well as its highly contagious nature. According to the WHO, there have been 223,022,538 cases and 4,602,882 deaths worldwide, while in Mexico, 3,465,171 cases and 265,420 deaths have been reported to date [2].

This worldwide contingency led to the development of various vaccines against SARS-CoV-2 to decrease the severity of cases and the need for hospitalization. Multiple vaccines are being deployed globally. There are currently 22 approved vaccines with different mechanisms of action promoting the development of spike-specific IgG antibodies with neutralizing capacity against SARS-CoV-2 [3]. The different designs of SARS-CoV-2 vaccines are messenger RNA vaccines such as BNT162b2 (Pfizer/BioNTech Inc., New York, NY, USA, and BioNTech SE, Mainz, Germany) and mRNA-1273 (Moderna TX, Inc., USA; Rovi Pharma Industrial Services S.A, Spain); adenoviral-vectored vaccines such as ChAdOx1 nCoV-19 (Oxford/AstraZeneca, AstraZeneca/SK Bioscience Co. Ltd., Republic of Korea; Serum Institute of India Pvt. Ltd., India; AstraZeneca AB, Sweden), Gam-COVID-VAC (Sputnik V, N.F. Gamaleya Ministry of Health of Rusia), Ad26.COV2 ( Janssen/Johnson & Johnson, Janssen–Cilag International NV, Belgium), and Ad5-nCoV (CanSinoBio, Tianjin, People’s Republic of China); protein subunit vaccines such as NVX-CoV2373 (Novavax SII, India), CoVLP (Medicago, Quebec, Canada) and GIGB66 (Abdala, Centro de Ingeniería Genética y Biotecnología, Cuba ); whole-cell inactivated virus vaccines such as CoronaVac (Sinovac, Life Sciences, LTD. China), BBIBP-CorV (Sinopharm, Beijing Institute of Biological Products Co. Ltd.) and Covarix (Bharat Biotech, India); finally DNA vaccines such as INO-4800 (INOVIO, California, USA) and ZyCoV-D (Zydus Cadila, Ahmedabad, India) [4,5].

In Mexico, ten vaccines against SARS-CoV-2 have been approved: (1) BNT162b2, (2) ChAdOx1 nCoV-19, (3) Ad5-nCoV, (4) CoronaVac (5) mRNA-1273, (6) Ad26.COV2.5, (7) Gam-COVID-VAC; (8) Covarix, and (9) BBIBP-CorV and (10) CIGB66 [6]. These vaccines were distributed following the national vaccination campaign guidelines.

The CanSinoBio vaccine, hereafter referred to as Ad5-nCOV, is an adenovirus type 5 vectored SARS-CoV-2 vaccine. It was developed in China as a single-dose vaccine, and has been approved in nine countries [7]. The phase III trials showed 68.83% and 95.47% efficacy against all symptomatic infections and severe disease 14 days after vaccination, respectively [8]. The Pfizer/BioNTech product, hereafter referred to as BNT162b2, is an mRNA vaccine that requires two doses with a 21-day period between each dose. The vaccine provides 95% effectiveness against SARS-CoV-2 infection in patients with both vaccines [9]. It has been approved in 122 countries, and the WHO recommends its application worldwide [10].

Currently, the new subject of interest is heterologous vaccine regimens against SARS-CoV-2. In a comparative study conducted in Germany, an increased immune response was observed in patients that used the ChAdOx1 nCoV-19 vaccine as a prime dose and BNT162b2 as a booster, in comparison with subjects who received the homologous regimen [11]. In a non-inferiority study conducted in the UK by Xinxue liu et al. in volunteers that could receive ChAdOx1/ChAdOx1, ChAdOx1/BNT162b2, BNT162b2/BNT162b2, or BNT162b2/ChAdOx1, and in which the time between the prime dose and the booster was 28 or 84 days, the heterologous schedules proved to be non-inferior to the homologous (ChAdOx1 nCoV-19/ChAdOx1 nCoV-19) in terms of the geometric mean ratio (GMR) of serum SARS-CoV-2 anti-spike IgG concentration (measured by ELISA), and no serious Adverse events following immunization (AEFI) were reported; this suggests flexibility in the use of heterologous prime-booster vaccinations. These results underscore the importance of obtaining information on other heterologous protocols [12]. However, further studies are necessary to address the safety and efficacy of other heterologous vaccine combinations.

In Mexico, the population that received the Ad5-nCoV vaccine was concerned about its efficacy. As a result, they started obtaining BNT162b2 boosters, against medical advice. In the second half of 2021, the Hospital Clínica Nova (HCN), in northern Mexico, began investigating the effect of SARS-CoV-2 vaccines. This study aimed to evaluate the effect of a single dose BNT162b2 boost in individuals after a completed Ad5-nCoV vaccination regiment compared to a group without this boost measuring SARS-CoV-2 Spike 1–2 IgG antibodies in plasma. We hypothesized that patients who received the BNT162b2 booster would have higher antibody concentrations in the 21–28 days of follow-up, and with no severe adverse reactions.

## 2. Material and Methods

This was a retrospective, prolective, and observational study that followed the STROBE guidelines at HCN [13]. The study included an analysis of a subgroup of patients who received the Ad5-nCoV immunization during the first trimester of 2021 in Monterrey, Nuevo León, Mexico. A larger prospective study has now been conducted, and a small subgroup that diverged from the protocol has been further analyzed. The study was approved by the local Institutional Review Board (Ref.:26022021-CN-1e-CI) and conducted per The Code of Ethics of the World Medical Association (Declaration of Helsinki) for human experiments. Due to the prospective nature of the study, all patients had to sign an informed consent form to participate.

The inclusion criteria were individuals of both genders, between the ages of 18 and 100 years, who had signed the informed consent form, and planned to complete the immunization regimen of any vaccine provided by the Mexican National Health System, and in compliance with the current immunization regimens. The criteria to exclude patients were if the time range of interest in this study had concluded or if they had previously received a SARS-CoV-2 vaccine.

The research team contacted the patients for an informative session on the protocol. After explaining the study to each patient, emphasizing that follow-up would last an entire year by determining SARS-CoV-2 specific IgG antibody concentrations in blood samples; all participants provided written informed consent. After reading it and agreeing to be part of the study, the phlebotomists collected a baseline plasma sample before vaccination.

The Mexican National Health System established that the vaccination campaign against SARS-CoV-2 would be divided according to age groups or occupation. Therefore, 21–28 days after receiving the first dose, the research team contacted the participants to collect the second plasma sample for IgG antibody determination. Three months after vaccination, a third sample was obtained, and subsequent sample collections were scheduled six and twelve months after the Ad5-nCoV regimen.

Every time the participants provided a sample for antibody determinations, they had to answer a questionnaire. The baseline-sample questionnaire was intended to obtain each patient’s medical history and record previous SARS-CoV-2 infections. The questionnaires applied after the first and second dose of the vaccine and aimed to recognize AEFI and identify a SARS-CoV-2 infection after receiving any vaccine dose [14,15]. After completing the vaccine regimen, the follow-up questionnaires on the fourth, fifth, and sixth IgG antibody sampling appointments, patients were questioned on any suspicious or confirmed SARS-CoV-2 infections.

Plasma was obtained by collecting 10 to 15 mL of blood by venipuncture. The sample was placed in tubes with ethylenediaminetetraacetic acid (EDTA) as an anticoagulant and stored at −80 °C. The laboratory personnel used the LIAISON SARS-CoV-2 S1/S2 IgG antibody detection kit, by Diasorin (Italy), to analyze the samples. It is based on chemiluminescence immunoassay (CLIA) technology to determine the amount of specific anti-S1 and anti-S2 IgG antibodies against SARS-CoV-2 in plasma samples. Its sensitivity was 97.4% (95% CI, 86.8–99.5) and its specificity was 98.5% (95% CI, 97.5–99.2). The results were reported as follows: <12.0 AU/mL was considered negative, 12.0 to 15.0 AU/mL was indeterminate, and >15 AU/mL was positive. The positive agreement with neutralizing antibodies was 94.4% [16,17].

In the three-month follow-up, patients who received a complete scheme of Ad5-nCoV were concerned about the effectiveness of a single-dose vaccine, so of their own volition, they received in this timeline a BNT162b2 booster, and they reported it to the research team. Additionally, patients were consented to get another plasma sample that was collected from the entire Ad5-nCoV recruited group 21–28 days after applying BNT162b2 (about 4 months of Ad5-nCoV dose). The inclusion criteria for the out of protocol analysis was to include all subjects that had received AD5-nCoV in the original protocol that accepted to get an extra blood sample 21–28 days after BNT162b2 boost.

We analyzed the following variables: age, gender, the time between the first and second vaccine, the medical history including previous diabetes or prediabetes according to ADA criteria [18], blood hypertension according to International Society of Hypertension [19], obesity (Body Mass Index > 30 kg/m^2^), dyslipidemia according to the American Association of Clinical Endocrinologist and American College of Endocrinology criteria [20], hypothyroidism, active cancer (any), smoking, and breastfeeding. A SARS-CoV-2 diagnosis was confirmed with a nasal swab or serologic (SARS-CoV-2 Spike 1–2 IgG antibodies) test before vaccination and through the entire study follow-up period. We analyzed AEFI caused by the Ad5-nCoV and BNT162b2 boost. The specific anti-S1 and anti-S2 IgG antibodies against SARS-CoV-2 were obtained before vaccination (S1), 21–28 days post-Ad5-nCoV (S2), three-months (follow-up) after Ad5-nCoV (S3), and 21–28 days after the BNT162b2-booster (S4).

The researchers reviewed the quality control and the anonymization of the database. Normality assumption was evaluated with the Shapiro-Wilk test and frequency histograms. Descriptive statistics such as median, the interquartile range for quantitative variables, and frequencies and percentages for categorical variables, were computed. The Kruskal–Wallis test was used for group comparison. The statistical program used was SPSS, version 2. The analysis was two-tailed. A *p*-value < 0.05 was considered statistically significant.

## 3. Results

There were 17 recruited participants immunized with Ad5-nCoV, 8 of whom received an additional BNT162b2 dose. We divided the data into four groups: (G1) patients vaccinated with complete Ad5-nCoV regiment with no history of SARS-CoV-2 infection (*n* = 4), (G2) patients vaccinated with complete regiment of Ad5-nCoV and the BNT162b2 boost, with no history of SARS-CoV-2 infection (*n* = 6), (G3) patients vaccinated with complete regiment of Ad5-nCoV with a history of SARS-CoV-2 infection (*n* = 5), and (G4) patients vaccinated with complete regiment of Ad5-nCoV with the BNT162b2 boost, with a history of SARS-CoV-2 infection (*n* = 2).

The patients mean (SD) age was 40 (16) years (G1 34 (15), G2 44 (6), G3 43 (9), and G4 44 (13)) and most participants were male (*n* = 9, 52.9%). Five patients had confirmed SARS-CoV-2 infection by PCR test before immunization, and two patients were positive after the first dose of AD5-nCoV and before the BNT162b2 booster. The time between complete regiment of Ad5-nCoV and the BNT162b2 boost was 93 (5) days. The most frequently reported comorbidity was obesity (*n* = 7, 41.2%), followed by hypertension (*n* = 3, 17.6%). Table 1 shows the participants’ medical history in the baseline sample questionnaire.

Data are presented as frequencies (percentage). NAFLD = non-alcoholic fatty liver disease. (G1) patients vaccinated with complete Ad5-nCoV regiment with no history of SARS-CoV-2 infection (G2) patients vaccinated with complete regiment of Ad5-nCoV and the BNT162b2 boost, with no history of SARS-CoV-2 infection, (G3) patients vaccinated with complete regiment of Ad5-nCoV with a history of SARS-CoV-2 infection, and (G4) patients vaccinated with complete regiment of Ad5-nCoV with the BNT162b2 boost, with a history of SARS-CoV-2 infection.

**Figure 1:**
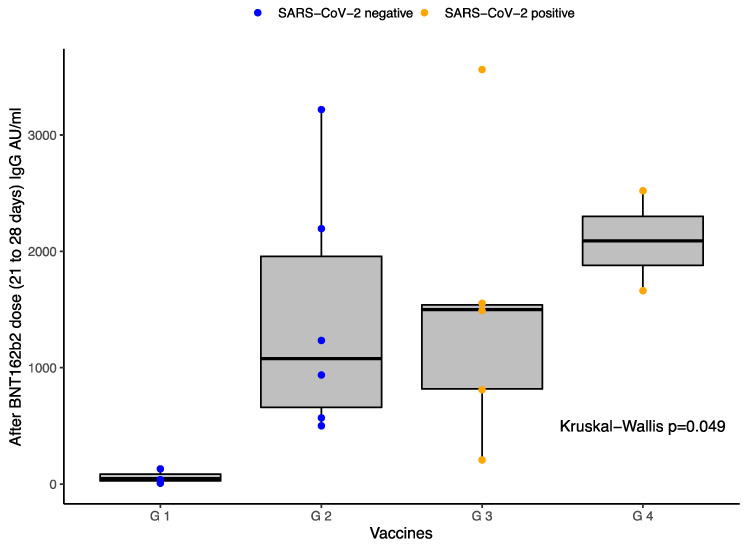
Anti-SARS-CoV-2 spike 1–2 IgG antibodies titers compared between groups.

The median baseline (IQR) values of the quantitative SARS-CoV-2 spike 1-2 IgG antibody titers obtained in S1 in the four groups were: 3.8 (0), 3.8 (0), 93 (231), and 3.8 (-) (*p* = 0.030), respectively. The results per group in S4 were: G1 46.7 (-), G2 1077.5 (1901), G3 1158.5 (2673.5), and G4 2090 (-) (*p* = 0.050). Table 2 shows the quantitative SARS-CoV-2 spike 1–2 IgG antibody titers against SARS-CoV-2 in the different groups of participants depending on the applied vaccines and SARS-CoV-2 history. Figure 1 also reports these results represented in a boxplot.

The boxplot in Figure 1 represents the IgG antibodies titers between the four different groups 21–28 days after BNT162b2 dose (about 4 months after first dose of Ad5-nCoV): G1 patients vaccinated with Ad5-nCoV with no known history of SARS-CoV-2 infection, G2 patients vaccinated with Ad5-nCoV and the first dose of BNT162b2 with no known history of SARS-CoV-2 infection, G3 patients vaccinated with Ad5-nCoV with a known history of SARS-CoV-2 infection, and G4 patients vaccinated with Ad5-nCoV and the first dose of BNT162b2 with a known history of SARS-CoV-2 infection. The boxplot shows the 25th, 50th, and 75th percentiles of antibody levels. The Kruskal–Wallis test was applied for comparisons showing a *p*-value = 0.049. The image shows statistical differences between all groups. Volunteers exposed to BNT162b2 after Ad5-nCoV have higher levels of IgG S1/S2 than those that did not receive the boost. Additionally, patients previously exposed to SARS-CoV-2 infection had higher antibody titers. The highest level of antibody levels was G4. Blue dots represent negative history of SARS-CoV-2 infection. Yellow dots represent positive history of SARS-CoV-2 infection. 

The most common AEFI related to AD5-nCoV were headache (*n* = 10, 83.3%), pain at the injection site (*n* = 8, 66.7%), and fatigue (*n* = 7, 58.3%), and their severity was classified as very low (*n* = 7, 58.3%). The most common AEFI reported after the BNT162b2 dose were pain at the puncture site (*n* = 5, 83.3%) and fatigue (*n* = 3, 50.0%), and their severity was classified as low (*n* = 3, 50.0%). Table 3 shows the AEFI related to the first dose of Ad5-nCoV and the BNT162b2 booster as reported by the participants at the time of the S3 and S4.

## 4. Discussion

This study compared the quantitative SARS-CoV-2 spike 1–2 IgG antibody titers after immunization with Ad5-nCoV, and after combining Ad5-nCoV and BNT162b2. The group immunized with a heterologous vaccine protocol had higher antibody titers and no serious adverse events when vaccines were applied ninety days apart. In addition, patients with a previous SARS-CoV-2 infection history had more elevated antibody titers as well.

Previous studies have compared the SARS-CoV-2 spike 1–2 IgG antibody titers after a single dose of an adenovirus-vector (ChAdOx1 nCoV-19) or an mRNA (BNT162b2 or mRNA-1273) vaccine, to analyze the efficacy of a one-dose vaccine. In their study, J. Spencer et al. reported that a single vaccine dose (either adenovirus-vector or mRNA) led to lower antibody titers than two doses of mRNA vaccine or a heterogeneous protocol with an adenovirus-vector and an mRNA vaccine [3]. Some authors agree that a heterologous vaccine regimen leads to a strong induction of antibodies. Such is the case of Tina Schmidt et al., who reported that IgG titers after an adenovirus-vectored vaccine and mRNA similar heterologous regimens were a significant improvement on the antibody titers, being approximately ten-fold higher than those obtained after homologous vector vaccination [21]. Our study is consistent with the increase in IgG levels in the group who followed a heterologous regimen. The groups that received a BNT162b2 booster had much higher antibody titers than those administered a single dose of an adenovirus-vector vaccine.

Studies on heterologous vaccination were started out of concern about the AEFI. For example, in March 2021, in Germany, the ChAdOx1 nCoV-19 vaccine administration was suspended due to concern over vaccine-induced cerebral venous thrombosis and thrombocytopenia syndrome, primarily in younger women [21]. This resulted in the implementation strategy of a second dose with an mRNA vaccine in individuals who had received an adenoviral-vector-vaccine as their first dose [21,22]. This recommendation was for people younger than 60 or 65 years in several European countries [23]. Powell et al. refers to this clinical advice because severe reactogenicity after the first dose is the most common reason for switching to a heterologous vaccine regimen [22]. However, in our study, the motivation to start a new vaccine schedule was due to the patients doubts on vaccine effectiveness. They did not receive any previous medical advice, and the decision was made on their own.

Nonetheless, adenovector-based vaccines such as Ad5-nCoV and ChAdOx1 nCoV-19 are not the only ones struggling with the AEFI rates. According to Qian He et al., mRNA vaccines, such as BNT162b2 Pfizer, had raised concerns due to the high incidence of AEFI, and this is why the heterologous regimen had been promoted. A study comparing a single-dose SARS-CoV-2 vaccine, priming with adenovector vaccines followed by inactivated vaccines, recombinant protein, or mRNA vaccines, concluded that sequential immunization with a heterogeneous regimen could potentially mitigate the AEFI of mRNA vaccines [24]. Tina Schmidt et al. compared the reactogenicity of a heterologous regimen with an mRNA booster. The results showed that both local and systemic AEFI were less severe and well-tolerated than those resulting from the homologous mRNA regimens [21].

We evaluated the AEFI and their perception. There were more AEFI reported after the Ad5-nCoV than after the BNT162b2 vaccine. The most frequently reported AEFI with Ad5-nCoV were headache, fatigue, and pain at the injection site, and their severity was mainly classified as very low, similar to that previously reported [25]. On the other hand, the number of AEFI reported with the BNT162b2 booster shot was lower, the most frequent being pain at the injection site, fatigue, and headache, and their severity perception was low. Our study concluded that the number of AEFI with the BNT162b2 booster is lower, but the perception of their severity was milder with Ad5-nCoV. Other studies have shown similar data, whereby heterologous schedules with a BNT162b2 booster led to more intense reactogenicity than in individuals who received the homologous-counterparts [25,26].

To the best of our knowledge, there are no previous studies in which one dose of Ad5-nCoV and a BNT162b2 booster have been studied. We believe that this study is valuable because it shows a good response in the quantitative SARS-CoV-2 spike 1–2 IgG antibody titers against SARS-CoV-2 with no severe AEFI. It also establishes the difference in antibody titers between patients with a history of SARS-CoV-2 infection, with higher detected antibody titers, as previously referred with other types of vaccines.

The approval of heterologous vaccination could be an opportunity to create more flexible vaccination programs, which is a significant issue in countries with scarce vaccine access, or in countries where different vaccines are available at different times. Additionally, protection against new SARS-CoV-2 variants must be considered [26]. Completing a two-dose regimen with the same vaccine could be challenging in cases of anaphylaxis or any severe reaction after the first dose, leading to the recommendation of a different vaccine as a second dose [22].

A limitation to this investigation is the sample size; however, we consider this information of value because of the robust follow-up of the patients and because to the best of our knowledge there is no information in relation to the combination of this type of vaccines. Further prospective studies must therefore recruit a more significant sample. Another limitation was the time interval between both vaccines. It could have more relevance if the time lapses were justified but it was established according to the patients’ programmed access to another vaccine.

## 5. Conclusions

This study analyzed a heterologous regimen based on BNT162b2 and Ad5-nCoV, a less commonly available vaccine. Patients receiving BNT162b2 after Ad5-nCoV had higher SARS-CoV-2 spike 1–2 IgG antibody titers and had no severe adverse reactions.

## Figures and Tables

**Table 1:** Medical history.

Medical Record (*n* = 17)	Frequency (%)	G1 (*n* = 4)	G2 (*n* = 6)	G3 (*n* = 5)	G4 (*n* = 2)
Pre-diabetes	2 (11.8)	0	1 (50)	1 (20)	0
Diabetes	0	0	0	0	0
Hypertension	3 (17.6)	0	1	2 (40)	0
Obesity	7 (41.2)	1 (25)	3 (50)	2 (40)	1 (50)
Dyslipidemia	1 (5.9)	0	0	1 (20)	0
Hypothyroidism	1 (5.9)	1 (25)	0	0	0
NAFLD	1 (11.8)	1 (25)	0	1 (20)	0
Active Smoker	2 (11.8)	0	0	0	2 (100)
Breastfeeding	1 (5.9)	0	0	1 (20)	0

**Table 2:** Quantitative SARS-CoV-2 spike 1-2 IgG antibody titers against SARS-CoV-2 in the different groups of participants depending on the applied vaccines and SARS-CoV-2 history.

Group (*n* = 17)	Before the First Shot of Vaccine (S1) (QIR)	21–28 Days Post Ad5-nCoV (S2) (QIR)	3 Months after Ad5-nCoV (S3) (QIR)	21–28 Days Post-BNT162b2 (S4) (QIR)
Ad5-nCoV/SARS-CoV-2 negative (G1) (*n* = 4)	3.8 (0)	49 (75)	54 (119)	46.7 (-)
Ad5-nCoV/BNT162b2/SARS-CoV-2 negative (G2) (*n* = 6)	3.8 (0)	62.25 (77)	28.6 (73)	1077.5 (1901)
Ad5-nCoV/SARS-CoV-2 positive (G3) (*n* = 5)	93 (231)	182 (3980)	1895 (4492)	1158.5 (2673.5)
Ad5-nCoV/BNT162b2/SARS-CoV-2 positive (G4) (*n* = 2)	3.8 (-)	61.05 (-)	35.56 (-)	2090 (-)
*p*-value *	0.030	0.313	0.028	0.049

Data are presented as medians (IQR). The Kruskal–Wallis test was applied for comparisons. Comparison between the initial and final values. * A *p*-value ≤ 0.05 was considered statistically significant.

**Table 3:** Adverse events in relation to vaccinations.

Adverse Events Following Immunization (AEFI)	Ad5-nCoV First Dose Frequency (%) (*n* = 17)	BNT162b2 Booster Frequency (%) (*n* = 8)
Presence of AEFI	12 (70.6)	6 (75.0)
Time lapse between the applied vaccine and the reported AEFI	First 4 h: 4 (23.5) Five to 24 h: 6 (35.6) 2 to 3 days: 2 (11.8)	First 4 h: 1 (16.7) Five to 24 h: 5 (83.3)
Headache	10 (83.3)	1 (16.7)
Pain at the injection site	8 (66.7)	5 (83.3)
Fatigue	7 (58.3)	3 (50.0)
Myalgia	3 (25.0)	0
Arthralgia	3 (25.0)	0
Insomnia	3 (25.0)	0
Edema at injection site	2 (16.7)	1 (16.7)
Nausea	2 (16.7)	0
Pruritus at puncture site	2 (16.7)	1 (16.7)
Fever	2 (16.7)	1 (16.7)
Low-grade fever	1 (8.3)	0
Adenopathy	1 (8.3)	0

Data are presented as frequencies (percentage). The percentages were calculated according to the number of patients that reported AEFI.

## Data Availability

Data are available upon request.

## References

[1] World Health Organization WHO Director-General’s Opening Remarks at the Media Briefing on COVID-19—11 March 2020. https://www.who.int/director-general/speeches/detail/who-director-general-s-opening-remarks-at-the-media-briefing-on-covid-19---11-march-2020.

[2] World Health Organization WHO Coronavirus (COVID-19) Dashboard. https://covid19.who.int.

[3] Spencer A.J., McKay P.F., Belij-Rammerstorfer S., Ulaszewska M., Bissett C.D., Hu K., Samnuan K., Blakney A.K., Wright D., Sharpe H.R. (2021). Heterologous Vaccination Regimens with Self-Amplifying RNA and Adenoviral COVID Vaccines Induce Robust Immune Responses in Mice. Nat. Commun..

[4] Sadarangani M., Marchant A., Kollmann T.R. (2021). Immunological Mechanisms of Vaccine-Induced Protection against COVID-19 in Humans. Nat. Rev. Immunol..

[5] Fathizadeh H., Afshar S., Masoudi M.R., Gholizadeh P., Asgharzadeh M., Ganbarov K., Köse Ş., Yousefi M., Kafil H.S. (2021). SARS-CoV-2 (COVID-19) Vaccines Structure, Mechanisms and Effectiveness: A Review. Int. J. Biol. Macromol..

[6] McGill COVID19 Vaccine Tracker Team Mexico—COVID19 Vaccine Tracker. https://covid19.trackvaccines.org/country/mexico/.

[7] McGill COVID19 Vaccine Tracker Team CanSino: Ad5-NCoV—COVID19 Vaccine Tracker. https://covid19.trackvaccines.org/vaccines/2/.

[8] Díaz-Ortega J.L., Pérez-Olguín J.E., Zuñiga-Ocampo C.O., Leriche-Ramírez C., Gaertner-Barnad S., Rodríguez-Paz M. Guía Técnica Para La Aplicación de La Vacuna Recombinante Contra El Nuevo Coronavirus (Vector de Adenovirus Tipo 5) de CanSino Biologics, Contra El Virus SARSCoV-2. https://coronavirus.gob.mx/wp-content/uploads/2021/03/GTApp_Cansino_16Mar2021.pdf.

[9] CDC Information about the Pfizer-BioNTech COVID-19 Vaccine. https://www.cdc.gov/coronavirus/2019-ncov/vaccines/different-vaccines/Pfizer-BioNTech.html.

[10] McGill COVID19 Vaccine Tracker Team Pfizer/BioNTech: BNT162b2—COVID19 Vaccine Tracker. https://covid19.trackvaccines.org/vaccines/6/.

[11] Tenbusch M., Schumacher S., Vogel E., Priller A., Held J., Steininger P., Beileke S., Irrgang P., Brockhoff R., Salmanton-García J. (2021). Heterologous Prime–Boost Vaccination with ChAdOx1 NCoV-19 and BNT162b2. Lancet Infect. Dis..

[12] Liu X., Shaw R.H., Stuart A.S.V., Greenland M., Aley P.K., Andrews N.J., Cameron J.C., Charlton S., Clutterbuck E.A., Collins A.M. (2021). Safety and Immunogenicity of Heterologous versus Homologous Prime-Boost Schedules with an Adenoviral Vectored and MRNA COVID-19 Vaccine (Com-COV): A Single-Blind, Randomised, Non-Inferiority Trial. Lancet.

[13] Cuschieri S. (2019). The STROBE Guidelines. Saudi J. Anaesth..

[14] Chen M., Yuan Y., Zhou Y., Deng Z., Zhao J., Feng F., Zou H., Sun C. (2021). Safety of SARS-CoV-2 Vaccines: A Systematic Review and Meta-Analysis of Randomized Controlled Trials. Infect. Dis. Poverty.

[15] Fan Y.-J., Chan K.-H., Hung I.F.-N. (2021). Safety and Efficacy of COVID-19 Vaccines: A Systematic Review and Meta-Analysis of Different Vaccines at Phase 3. Vaccines.

[16] Bonelli F., Sarasini A., Zierold C., Calleri M., Bonetti A., Vismara C., Blocki F.A., Pallavicini L., Chinali A., Campisi D. (2020). Clinical and Analytical Performance of an Automated Serological Test That Identifies S1/S2-Neutralizing IgG in COVID-19 Patients Semiquantitatively. J. Clin. Microbiol..

[17] DiaSorin’s LIAISON® SARS-CoV-2 Diagnostic Solutions. https://www.diasorin.com/en/immunodiagnostic-solutions/clinical-areas/infectious-diseases/covid-19.

[18] American Diabetes Association 2 (2020). Classification and Diagnosis of Diabetes: Standards of Medical Care in Diabetes—2021. Diabetes Care.

[19] Unger T., Borghi C., Charchar F., Khan N.A., Poulter N.R., Prabhakaran D., Ramirez A., Schlaich M., Stergiou G.S., Tomaszewski M. (2020). 2020 International Society of Hypertension Global Hypertension Practice Guidelines. Hypertension.

[20] Handelsman Y., Jellinger P.S., Guerin C.K., Bloomgarden Z.T., Brinton E.A., Budoff M.J., Davidson M.H., Einhorn D., Fazio S., Fonseca V.A. (2020). Consensus Statement by the American Association of Clinical Endocrinologists and American College of Endocrinology on the Management of Dyslipidemia and Prevention of Cardiovascular Disease Algorithm—2020 Executive Summary. Endocr. Pract..

[21] Schmidt T., Klemis V., Schub D., Mihm J., Hielscher F., Marx S., Abu-Omar A., Ziegler L., Guckelmus C., Urschel R. (2021). Immunogenicity and Reactogenicity of Heterologous ChAdOx1 NCoV-19/MRNA Vaccination. Nat. Med..

[22] Powell A.A., Power L., Westrop S., McOwat K., Campbell H., Simmons R., Ramsay M.E., Brown K., Ladhani S.N., Amirthalingam G. (2021). Real-World Data Shows Increased Reactogenicity in Adults after Heterologous Compared to Homologous Prime-Boost COVID-19 Vaccination, March−June 2021, England. Eurosurveillance.

[23] Normark J., Vikström L., Gwon Y.-D., Persson I.-L., Edin A., Björsell T., Dernstedt A., Christ W., Tevell S., Evander M. (2021). Heterologous ChAdOx1 NCoV-19 and MRNA-1273 Vaccination. N. Engl. J. Med..

[24] He Q., Mao Q., An C., Zhang J., Gao F., Bian L., Li C., Liang Z., Xu M., Wang J. (2021). Heterologous Prime-Boost: Breaking the Protective Immune Response Bottleneck of COVID-19 Vaccine Candidates. Emerg. Microbes Infect..

[25] Shaw R.H., Stuart A., Greenland M., Liu X., Nguyen Van-Tam J.S., Snape M.D., Com-COV Study Group (2021). Heterologous Prime-Boost COVID-19 Vaccination: Initial Reactogenicity Data. Lancet.

[26] Duarte-Salles T., Prieto-Alhambra D. (2021). Heterologous Vaccine Regimens against COVID-19. Lancet.

